# Study draft: “UVC—You Will See” study: longer vs. shorter umbilical venous catheter (UVC) dwell time (6–10 vs. 1–5 days) in very premature infants with birth weight < 1250 g and/or gestational age < 30 weeks

**DOI:** 10.1007/s10354-024-01047-7

**Published:** 2024-06-13

**Authors:** Sascha Meyer, Steffi Hess, Martin Poryo, Cihan Papan, Arne Simon, Silvia Welcker, Anne Ehrlich, Christian Ruckes

**Affiliations:** 1 Department of Pediatrics and Neonatology, University Hospital Saarland, Homburg, Germany; 2 Kinder- und Jugendmedizin, Klinikum Saarbrücken Winterberg, Saarbrücken, Germany; 3 Department of Pediatric Cardiology, Saarland University Medical Center, Homburg, Germany; 4 Institute for Hygiene and Public Health, University Hospital Bonn, Bonn, Germany; 5 Department of Pediatric Hematology and Oncology, and Infectious Diseases, Saarland University Medical Center, Homburg, Germany; 6 Franz-Lust Klinik für Kinder und Jugendliche, Städtisches Klinikum Karlsruhe, Moltkestraße 90, 76135 Karlsruhe, Germany; 7 Interdisziplinäres Zentrum für Klinische Studien (IZKS), Mainz, Germany

**Keywords:** Premature infants, Central venous access, Catheter-associated complications, Pain, Outcome, Frühgeborene, Zenralvenöse Katheter, Katheter-assoziierte Komplikationen, Schmerzen, Outcome

## Abstract

**Background:**

Umbilical venous catheters (UVCs) are often used in preterm infants. Their use is associated with complications (infections, clot formation, organ injury). Very preterm infants with acquired bloodstream infection are at a higher risk for death and important morbidities (e.g., adverse neurodevelopmental outcomes). It is standard clinical practice to remove UVCs in the first days of life. Replacement of intravenous access is often performed using percutaneously inserted central catheters (PICCs). It is unclear whether serial central line use affects the rates of catheter-related complications.

**Methods:**

A multicenter randomized controlled trial (random group assignment) was performed in 562 very premature (gestational age < 30 weeks) and/or very low birth weight infants (< 1250 g) requiring an UVC for administration of parenteral nutrition and/or drugs. Group allocation was random.

**Hypothesis:**

A UVC dwell time of 6–10 days (281 infants) is not associated with an increased rate of central venous catheter (UVC, PICC)-related complications compared to 1–5 days (281 infants), and a longer UVC dwell time will significantly reduce the number of painful, invasive procedures associated with the need for vascular access as well as radiation exposure, use of antibiotics, and medical costs.

**Primary outcome parameter:**

The number of catheter-related bloodstream infections and/or catheter-related thromboses and/or catheter-associated organ injuries related to the use of UVC/PICC was the primary outcome.

**Conclusion:**

Extending the UVC dwell time may significantly reduce the number of painful invasive procedures, with the potential to positively impact not only long-term pain perception but also important social competencies (attention, learning, and behavior). Thus, the “UVC—You Will See” study has the potential to substantially change current neonatal intensive care practice.

## Abstract (de)

**Hintergrund:**

Nabelvenenkatheter (NVK) werden bei Frühgeborenen zur künstlichen Ernährung und Medikamentengabe verwendet. Sie sind allerdings mit Komplikationen verbunden (Infektionen, Blutgerinnsel, Organverletzungen). Sehr unreife Frühgeborene mit erworbenen Infektionen der Blutbahn weisen ein höheres Risiko für Tod und wesentliche Morbidität auf (z. B. ungünstige neurologische Entwicklung). Es ist daher gängige klinische Praxis, NVK bereits in den ersten Lebenstagen zu entfernen. In der Regel werden daran anschließend peripher eingeführte zentralvenöse Katheter verwendet (PICC). Dies bedeutet einen weiteren schmerzhaften und möglicherweise mit Komplikationen einhergehenden Eingriff. Bis heute ist der ideale Zeitpunkt für die NVK-Entfernung nicht geklärt.

**Methoden:**

Es handelt sich um eine multizentrische Studie mit 562 sehr unreifen Frühgeborenen (Gestationsalter < 30 Schwangerschaftswochen) und/oder einem Geburtsgewicht < 1250 g, bei denen ein NVK zur Gabe von parenteraler Ernährung und/oder Medikamenten notwendig war. Die Gruppenzuteilung erfolgt nach dem Zufallsprinzip.

**Fragestellung:**

Eine NVK-Liegedauer von 6–10 Tagen (281 Frühgeborene) verglichen mit einer von 1–5 Tagen (Frühgeborene) ist nicht mit einer erhöhten Rate an katheterassoziierten (NVK, PICC) Infektionen, Blutgerinnseln oder Organverletzungen verbunden. Eine verlängerte NVK-Liegedauer führt zu weniger schmerzhaften Anlagen von Gefäßzugängen, zu einer verringerten Strahlenbelastung (Röntgenuntersuchungen), zu einem verminderten Gebrauch von Antibiotika sowie zu einer Kostenreduktion.

**Primäre Zielvariable:**

Primär untersucht wurden Unterschiede bzgl. der Anzahl der durch zentralvenöse Katheter (NVK/PICC) bedingten Komplikationen.

**Schlussfolgerung:**

Die Verlängerung der NVK-Liegedauer führt möglicherweise zu einer deutlichen (signifikanten) Verringerung der schmerzhaften Eingriffe, Strahlenbelastung, Verwendung von Antibiotika sowie Kostenreduktion ohne Erhöhung von Komplikationen. Dies kann sich nicht nur auf die langfristige Schmerzwahrnehmung auswirken, sondern hat auch einen positiven Effekt auf die Gesamtentwicklung, z. B. soziale Kompetenzen (Aufmerksamkeit, Lernen, Verhalten). Die Studie „UVC—You Will See“ hat das Potenzial, die Behandlung von sehr/extrem unreifen Frühgeborenen nachhaltig zu verändern.

## Background

### Medical problem

Umbilical venous catheters (UVCs) are commonly used to establish central vascular access for delivery of parenteral nutrition and drugs to preterm and/or sick newborn infants [1, 2]. They were first introduced into clinical practice some 50 years ago [3]. Evidence suggests that use of UVCs rather than peripheral venous cannulas facilitates consistent delivery of parenteral nutrients and reduces the number of painful venipunctures [4, 5]. Because UVCs terminate within the inferior vena cava, their use may reduce the risk of subcutaneous extravasation injury caused by hyperosmolar solutions and medications [6]. Alternatively, or in addition to UVC, percutaneously inserted central lines may be used (PICCs) [7].

### Prevalence, incidence, mortality

The use of UVCs is associated with complications like infections, thrombosis, malposition, and organ injury [8–13], with bloodstream infection being the most common serious adverse event (incidence: 3–> 20%) [14–19]. In particular very low birth weight (VLBW) infants with acquired bloodstream infection are at a higher risk for mortality and important morbidities (bronchopulmonary dysplasia [BPD]; necrotizing enterocolitis [NEC]; retinopathy of prematurity [ROP]) and prolonged hospitalization [20] as well as adverse neurodevelopmental outcomes [21–23]. Ongoing controversy surrounds the duration of placement [2–18, 18–25]. The US Centers for Disease Control and Prevention (CDC) recommends that UVCs should be removed as soon as possible when no longer needed but can be used for up to 14 days if managed aseptically [16]. Based on published data in the medical literature [16–19], results from our survey of participating centers in the “UVC—You Will See Study” [26] as well as data from our “UVC—You Will See Pilot Study” (DRKS-ID: DRKS00022262; [27]), we assume an event rate of central venous catheter-related adverse events (UVC/PICC) of 30%.

### Burden of disease

Consistent with these recommendations, standard clinical practice in many NICUs is often to remove UVCs in the first days of life. Replacement of intravenous access is often performed using PICCs or peripheral cannulas [13]. It is unclear whether this strategy of serial central line use affects rates of catheter-related complications [14, 15]. During their NICU stay, VLBW/very low gestational age neonates (VLGAN) are subjected to numerous painful procedures [5]. The most significant long-term clinical effects of early pain exposure may contribute to later attention, learning, and behavioral problems [28]. Thus, reducing the number of painful invasive procedures has the potential to positively impact not only long-term pain perception but also important social competencies.

## Improvement of therapy/impact of the trial

### Novelty

The “UVC—You Will See” study will address primarily the effects of later removal (6–10 days dwell time) vs. early planned removal (1–5 days dwell time) on the risk of central venous catheter-related bloodstream infections, thrombosis, and organ injury in a cohort of very premature infants, and will provide the neonatal community with robust new data with regard to the optimal UVC dwell time.

### Clinical impact/patient benefit

The “UVC—You Will See” trial has the potential to significantly alter the treatment of this highly susceptible cohort, i.e., by reducing the number of CVC catheters, reducing the number of painful invasive procedures related to vascular access, reducing radiation exposure, reducing the use of antibiotics, and reducing medical expenditures without putting these infants at an increased risk of CVC-associated complications. In the long run, the most significant clinical effects of early pain exposure may be on neurodevelopment, contributing to later attention, learning, and behavioral problems in these vulnerable children [28]. Thus, reducing the number of painful invasive procedures has the potential to positively affect both long-term pain perception as well as important social competencies.

### Socioeconomic impact

This study has the potential to significantly reduce the number of CVCs used—both UVCs and PICCs—as well as the number of radiographs and the use of antibiotics, as demonstrated in our pilot trial (DRKS00022262; [27]). Also, reducing the long-term burden of very and extreme prematurity by improving social competencies has the potential to reduce medical expenditures.

### Patient and target group involvement plan

The “UVC” study has received full endorsement by the German patient advocacy group *Bundesverband: Das Frühgeborene*. They were particularly interested in reducing the number of painful invasive procedures with regard to vascular access without unduly increasing the risk of CVC-related complications. We also agreed on further 6‑monthly consultations with *Bundesverband: Das Frühgeborene* during the “UVC—You Will See” study, thus guaranteeing regular face-to-face assessment and modifications as deemed necessary. In summary, this RCT will address key clinical aspects in the care of very and extremely premature infants with both short- and long-term implications in cooperation with *Bundesverband: Das Frühgeborene*.

### Evidence: results from the literature search/own pilot study and survey

Articles were identified by searching Medline, Embase, Web of Science, the Cochrane Library databases, clinicaltrials.gov, German Clinical Trials Register (“*Deutsches Register Klinische Studien*” [DRKS]), and the International Clinical Trials Search Portals (ICTRP) for peer-reviewed, English-language articles published from 1990 through to 2023. We included all relevant references from the literature search (and focused on the Cochrane review by Gordon et al.; [29]). All relevant studies pertinent to this topic listed in the Cochrane meta-analysis, most importantly RCTs, were analyzed in detail. We analyzed all publications with regard to adverse side effects associated with the use of UVCs and PICCs (see Appendix A). Moreover, we used data from our current pilot RCT (DRKS 00022262; [27]) to estimate the rate of catheter-related complications and to provide guidance with regard to the best time intervals (“dwell times”). Based on these findings and those from our questionnaire/survey of all participating centers (*n* = 13) [26], we decided to compare a dwell time of 1–5 days vs. 6–10 days with an overall estimated event rate of CVC-related complications—both UVC-/PICC-related—of 30%.

### Justification of design aspects

There is a paucity of clinical data from high-quality studies (i.e., RCTs) that addressed the important issue of optimal UVC dwell times. Given the potential for benefit and harm associated with the timing of removal of UVCs, a multicenter, randomized controlled trial in infants with a birth weight < 1250 g (≥ 500 g) and/or a gestational age < 30 weeks (≥ 24 weeks of gestation) is warranted to provide robust data on this issue. This highly susceptible cohort was chosen because the vast majority of these infants will initially require prolonged central venous access (e.g., UVC/PICC). Thus, the RCT “UVC—You Will See” trial has the potential to significantly alter current treatment of these patients.

## Methods

### Inclusion/exclusion criteria

In this RCT, we will enroll very and extremely premature and very and extremely low birth weight infants (birth weight < 1250 g and/or a gestational age < 30 weeks) who require insertion of an UVC for parenteral nutritional and/or drug administration because this represents the “standard population” of premature infants that will most commonly require prolonged central vascular access.

### Intervention(s)

The “UVC—You Will See Study” is a pragmatic RCT comparing two different dwell times within the manufacturer’s recommendations. While the manufacturers’ specifications allow dwell times of up to 14 days, it is common clinical practice to remove UVCs in most NICUs within the first days of life. Moreover, data from our pilot RCT “UVC—You Will See Study” indicate that a dwell time > 10 days is associated with an increased rate of UVC-associated complications (unpublished data).

### Outcome measures

We chose *primary, key secondary, and secondary endpoints, as well as an exploratory endpoint* since it is considered the “gold standard” in the assessment of catheter-related complications. Importantly, in doing so, we will also assess potential benefits associated with a longer dwell time (i.e., fewer painful invasive procedures for vascular access, less radiation exposure, fewer days of antibiotics, reduction in medical expenditures). BPD, NEC, FIP, IVH, PVL, and ROP comprise all major neonatal morbidities, and mortality will be assessed.

#### Primary efficacy endpoint

Number of catheter-related bloodstream infections and/or catheter-related thromboses/emboli and/or catheter-associated organ injuries including cardiac arrhythmias related to the use of UVC and/or peripherally inserted central catheters (PICC).

#### Key secondary endpoint(s)

Number of catheter-related bloodstream infections and/or catheter-related thromboses/emboli and/or catheter-associated organ injuries including cardiac arrhythmias related to the use of UVC.

#### Secondary endpoints

Number of painful procedures associated with insertion of UVC, PICC, and peripheral catheters; number of X‑rays for assessment of correct placement of UVC/PICC (radiation exposure); use of antibiotics with regard to suspected/proven CVC-associated (UVC/PICC) bloodstream infection; medical costs associated with the central venous catheters (UVC and PICC).

#### Exploratory endpoint

Correlation between length of dwell time and primary outcome parameter. *Assessment of safety:* Standardized clinical and ultrasonographic assessment as per study protocol; additional septic work-up as indicated as well as electrocardiography (ECG) examination in case of cardiac arrhythmia.

### Methods against bias

This trial will be conducted as a multicenter, active-controlled RCT. Neonates with a birth weight < 1250 g and/or a gestational age < 30 weeks who require UVC for parenteral nutrition and/or drug administration will be randomized to either a catheter dwell time of 1–5 days (standard therapy) vs. a dwell time of 6–10 days (interventional therapy). A randomization list will be generated by the Interdisciplinary Centre for Clinical Trials (IZKS) Mainz. The randomization ratio will be 1:1 using block randomization. A web-based randomization tool developed by IZKS Mainz will be used in this trial, allowing investigators to randomize patients via a secure web interface. While randomization is feasible, blinding of treating physicians is not possible due to the nature of the intervention. However, assessment of major outcome parameters (e.g., number of painful procedures, radiation exposure, use of antibiotics, medical costs) will be assessed by an independent researcher blinded to dwell times.

### Proposed sample size/power calculations

**Fig. 1:**
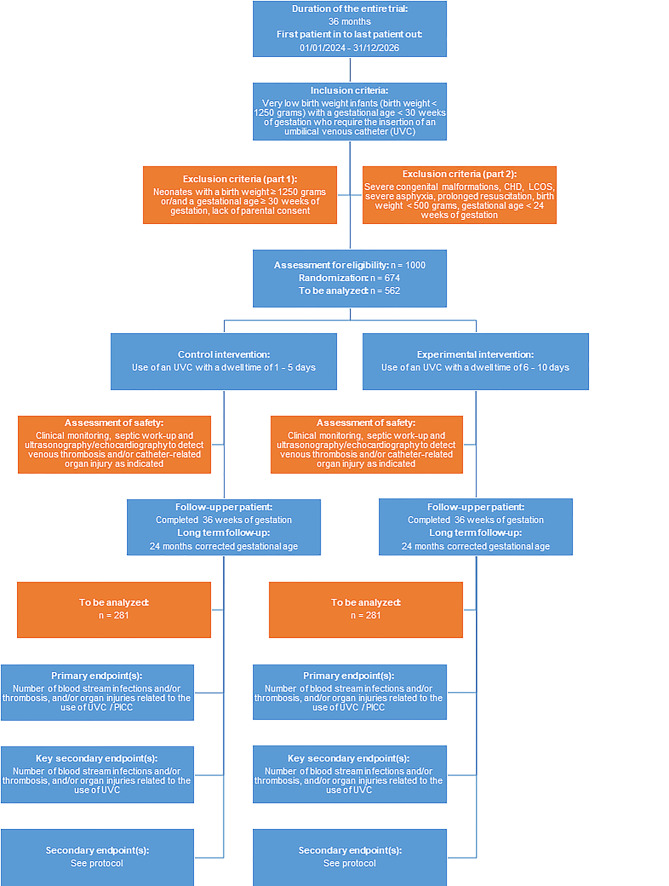
intervention scheme/trial flow

For the sample size calculation—based on an extensive literature review, a current survey of participating centers, and results from our pilot study—we assume an event rate of 30% in the control group. The non-inferiority bound was fixed to 10%. The sample size was planned with a one-sided level of significance of 5% and a power of 80% using a two-sample t‑test. Non-inferiority will be concluded if the one-sided 95% confidence interval of the treatment difference is located completely below the non-inferiority bound. The sample size amounts to 562 (= 2 × 281) evaluable patients. The primary analysis is the per protocol population consisting of all randomized patients complying sufficiently with the study protocol. When assuming 20% of the randomized patients will not be part of the per-protocol population, 674 patients will have to be randomized (Fig. 1). The sample size was calculated with SAS version 9.4 (Cary, NC, USA).

### Feasibility of recruitment

All participating centers are level-III NICUs with extensive expertise in the management of VLBW and ELBW infants. The participating centers have been actively involved in a number of large, multicenter neonatal trials, and have repeatedly demonstrated their competency in successfully recruiting adequate numbers of patients in a number of different trials (e.g., 12/14 centers actively participate in the NeoVitaA trial), and all 14 centers have provided full commitment to study participation and adequate patient enrollment. In our pilot single-center RCT “UVC—You Will See,” we enrolled 64 patients within an 18-month period. Thus, the recruitment of 562 infants by 14 level-III NICUs over a 3-year period is feasible. Additional centers will be contacted for potential study participation if required.

### Statistical analysis

The primary endpoint will be analyzed within a logistic regression model with treatment as the fixed factor and center as covariate. Non-inferiority will be concluded if the one-sided 95% confidence interval of the absolute risk difference of the experimental intervention and the control intervention is completely located below the non-inferiority bound of 10%. The primary analysis population is the per-protocol population consisting of all randomized patients who sufficiently comply with the protocol, i.e., all subjects without violation of any exclusion or inclusion criteria and with an UVC at least 1 or 6 days respectively. For the experimental intervention, we expect a UVC dwell time of 6–10 days in 90% of the patients. The population is chosen very liberally so as not to introduce any bias, e.g., by excluding informative dropouts. The results in the ITT population will be considered as equally important. Since the primary endpoint is composite, the analysis will be repeated for every single component. Several sensitivity analyses will be performed. Additional parameters like birth weight, gestational age, hematocrit, or gender will be included in the analysis. Descriptive statistics will be displayed whenever appropriate.

The key secondary endpoint will be analyzed by the same model as the primary analysis. However, the objective is to show superiority of the experimental treatment versus the control treatment. Other secondary endpoints will be analyzed by a negative binomial regression model. Although they are considered exploratory, they will be interpreted at a significance level of α = 5%.

## Ethics

The study protocol of our pilot study “UVC—You Will See” was approved by the ethics committee of the University of Saarland and by the Federal Institute for Drugs and Medical Devices (“*Bundesinstitut für Arzneimittel und Medizinprodukte*” [BfArM]), and is listed at German Clinical Trials Register. This study has the potential to demonstrate that a longer dwell time (6–10 days) is not associated with an increased rate of catheter-related complications, as shown in our pilot trial (DRKS-ID: DRKS00022262; unpublished data). Professor Dr. med. Sascha Meyer is an expert in the field and has successfully carried out a number of large clinical neonatal trials, e.g., the NeoVitaA trial (DFG; ME 3827/1-1/2). The IZKS Mainz is very experienced in the realization and implementation of large, multicenter RCTs in a variety of different medical fields, including neonatology (e.g., the NeoVitaA trial), thus guaranteeing a highly professional infrastructural framework. In doing so, all relevant aspects with regard to safety issues in this study will be covered, and continuous monitoring of participating centers will be provided by the IZKS Mainz.

## Data handling

Responsible for data handling is the IZKS Mainz. A validated trial data management system will be used for data capture, processing, and storage. Long-term electronic data storage is warranted. The applicants ensure that upon qualified request, trial data will be made available for meta-analyses, for disease-related registries, or any other future scientific reuse, if applicable. The FAIR principles will be adhered to.

## Appendix A

### Search strategy

Articles for the literature review were identified by searching Medline, Embase, Web of Science, the Cochrane Library databases, clinicaltrials.gov, German Clinical Trials Register (“*Deutsches Register Klinische Studien*” [DRKS]), and the International Clinical Trials Search Portals (ICTRP) for peer-reviewed, English-language articles published from 1990 through 2022. We performed a systematic literature review including the search terms “very low birth weight infant,” “painful, invasive procedures,” “umbilical venous catheter,” “dwell time,” “catheter-related bloodstream infection,” “thrombosis,” “organ injury.” For our search strategy, we used a combination of two or more of the abovementioned search terms, always including “very low birth weight infants.” The titles and abstracts of the retrieved citations were reviewed to determine which met the inclusion criteria outlined in the search strategy above (articles published in English, year of publication, published complete articles, human subjects, abstract available). While RCTs were regarded as of major importance, observation studies as well as reviews by known experts in the field were retrieved as well and included if considered of adequate quality in our literature analysis.

## Abbreviations

BPD: Bronchopulmonary dysplasia
CVC: Central venous catheter
ELBW: Extremely low birth weight
FIP: Focal intestinal dysplasia
IVH: Intraventricular hemorrhage
NEC: Necrotizing enterocolitis
NICU: Neonatal intensive care unit
PICC: Peripherally inserted central catheter
RCT: Randomized controlled trial
ROP: Retinopathy of prematurity
UVC: Umbilical venous catheter
VLBW: Very low birth weight
VLGAN: Very low gestational age
